# For an indeterministic ethics. The emptiness of the rule *in dubio pro vita *and life cessation decisions

**DOI:** 10.1186/1747-5341-4-6

**Published:** 2009-05-14

**Authors:** Dragan Pavlovic, Christian Lehmann, Michael Wendt

**Affiliations:** 1 Department of Anaesthesiology and Intensive Care Medicine, Ernst Moritz Arndt University, Greifswald, Germany

## Abstract

It is generally claimed that there exist exceptional circumstances when taking human life may be approved and when such actions may be justified on moral grounds. Precise guidelines in the medical field for making such decisions concerning patients who are terminally ill or have irreparable injuries incompatible with a bearable life, are difficult to establish. Recommendations that take the particular logical form of a rule, such as "in dubio pro vita", "when in doubt favour life") have been suggested and in some countries incorporated into legal texts (Germany). We claim here that such a rule is of no value since it is open-ended and always allows for doubt, and a decision to employ measures that would support human life could always be argued to be a valid choice. Preservation of this rule could be encouraged, but giving it the force of law may put physicians at risk, as they may be challenged for choosing to terminate life in otherwise ethically and medically uncontroversial circumstances.

## Background

Medical personnel can face extremely difficult choices when confronted with a patient for whom life saving or life prolonging measures do not seem justified. They are advised to then use a simple rule viz. *in dubio pro vita *– "when in doubt, favor life". The intention of this paper is to expose the concealed logical structure of the rule *in dubio pro vita*, demonstrate its theoretical shortcomings, and present hypothetical practical difficulties for using it not only as a "rule" but in particular as a "rule" having the force of law, in Germany, for example [1]. Our aim, therefore, is to explore ethical aspects of the logic of the rule as well as relevant legal interpretations. We will leaving aside issues which are largely discussed elsewhere, including those in basic ethical textbooks or books and articles on the clinical application of medical ethics.

Indeed, the question as to whether killing could be morally justified has a very long history. In modern times this problem arises when trying to justify ending the life of terminally ill patients – such patients now comprise the majority of intensive care units (ICU) deaths [2] – or of those with irreparable injuries incompatible with a bearable life. In practice a utilitarian approach to ending life has replaced a deontological approach which, in its strongest form, would forbid any termination of human life. Nonetheless, a practical and uncontroversial guide for deciding when to end someone's life is not available, even in situations involving patients who might be considered for life termination (some emergency or ICU patients) and who would certainly die from their condition. Furthermore, the routines, customs, limitations and practices vary substantially between countries [3], and the mere defining of a condition that would indisputably allow life termination cannot be agreed upon. Scholarly debate reflects the struggle to define such a life terminating condition [4-6], and debate persists as to whether life termination conditions even exist [7,8]. Consequently life termination can always be questioned, even with the most precise definitions. In principle, no form of consequentialism – which prescribes that our actions should be guided by their consequences – can provide a right answer for what ought to be done, since there is no certainty as to how various agents (people), whose behavior one cannot control, would behave [9-12]. As a result, uncertainty, no matter how minor in degree, remains. We will not, in this manuscript, further focus upon consequentialism, apart from the above-mentioned problem of calculation and the prediction of outcomes in a human discipline such as medicine. In natural sciences, such as physics, events are often highly predictable. On the other hand, when consequential calculations involve the prediction of social events (medical science may be of some special kind close to social sciences), our predictive power is very low [14-16]. Therefore, consequentialists' aims are hypothetical in principle. Indeed, for our actions to be justified by their ends (consequential logic), it must be certain not only that the ends are right and good but that they would be right if they increased the good [13], and furthermore that they occur with certainty. If they do not, we will then be basing our actions on mere intentions, i.e. predictions that may not ensue. It is then not the benefits of the outcome that justifies consequentialist actions but the intentions – and precisely here is where consequentialist morality fails. This weak point of consequentialism (and utilitarianism, which is a special case of consequentialism), was seen very early and by a number of critics, some of whom were quite famous, as, for example, in the late 19th century, Nietzsche, or more recently, Bernard Williams and John Rawls. Nietzsche, in his "The Will to Power," dating from 1888 [9], mentions utilitarianism and comments with doubt that consequences can be known. John Rawls repeatedly expresses concern in his "Theory of Justice" [10] about the difficulties of calculating utility and prediction in utilitarian arguments. Also, in his "Political Liberalism" [11], Rawls, referring to teleological theories of justice, states that: "... the form of public reasoning they specify tends to be politically unworkable..." and, referring to consequentialists calculations of benefits, remarks on the "...highly speculative nature and enormous complexity of these calculations..." Another moral philosopher who objects to utilitarianism, and specifically considers the problem of prediction of desired events is the above mentioned Bernard Williams (in the introductory paragraphs of the first and second section of his text) [12].

The following example may help the reader better appreciate the circumstances in medical practice. In serious situations, for example after a car accident with multiple trauma to thorax and extremities as well as head injury with basilar skull fractures, a physician may have to decide, in the face of considerable doubt about how to handle the situation, whether to continue life support or not. One might imagine that heroic, immediate maximal care – with extensive surgery and all possible imaginable medical measures – could be life saving for an unknown small number of these patients who would otherwise die. Often it is clear to the practicing emergency physician (whom we can describe as "skeptical") that all of this is in reality unavailable and that "there is no doubt" that the patient would be lost anyway – so in such cases further intensive life saving measures will not be administered. Exactly at that point an "optimist" may have a different opinion and this would be enough for the controversy to take on an ominous dimension. These cases exist in the practice of emergency medicine and in the ICU, but they almost never surface in public because of their extreme ambiguity which is fully appreciated by medical personnel.

In an effort to reduce uncertainty in practical patient care, some "precise" rules have been established. Ideally, decisions to continue or discontinue life should be reviewed by a collective of experts. However, it is maintained that expert opinion be based on a lengthy, elaborate education and years of training [17,18]. Medical personnel with various degrees of competence, experience and medical expertise are continually facing problems associated with terminal care [4,5,19] or with emergency care. Expert group consultation is not always possible, thereby creating a need for more simple and reliable life support guidelines. Yet, over-defining the rules for such situations, we will argue, may introduce the possibility of erroneous decisions. Introducing such rules into the law may even worsen the situation, since all that would be achieved would be the transfer of an ambiguity from an everyday life context to a judicial context.

To provide a practical rule, the last resort of consequentialism has been an adaptation of the rules prescribed in other fields. The rule "*in dubio pro reo*" ("when in doubt, favour the accused"), probably derived from Roman law, has been extended to environmental issues, for example, the rule "*in dubio pro natura*" ("when in doubt, favor nature") [18] and finally in medical ethics, to the rule *in dubio pro vita *("when in doubt, favor life"). Some European legislatures have gone so far as to incorporate *in dubio pro vita *as a legal term (Germany) [1,20-22]. As we will see in what follows, there are logical, epistemic and legal objections to the tendencies to give the aforementioned rule the force of law. The claim that we criticize is that the rule *in dubio pro vita *is of practical help to the medical personnel deciding about the cessation or continuation of life support. Quite to the contrary, we claim that incorporating the rule *in dubio pro vita *in positive law may offer false and dangerous security to medical practitioners when they are dealing with the terminally ill or untreatable severely injured patients. Alternatively, it may lead to unreasonable attempts to maintain life in these patients, since the rule may be interpreted as forbidding every termination of life support.

## Logic and reality of *in dubio pro vita*

When considering whether or not to support human life, the decision has to be based on morally acceptable reasons, including the patient's desires, together with existential reasons, including medical reasons such as absence of mental activity, certainty that life could not be prolonged, etc. If certainty is not there (i.e. doubt exists), *in dubio pro vita *prescribes that one has to favor life. Or similarly, if one is not in doubt but others are, whether a family member or a legal or moral authority, then some degree of doubt exists and one should again favour life. One should then, by acting or not acting, permitprolongation of life. Otherwise, if absolutely no doubt exists, and all important conditions are satisfied (including that the positive laws permit such acts), one may passively or even actively (quite exclusively in some countries) terminate life. We argue that, on grounds of formal logic, unfortunately, the cited "rule" is not a useful "rule" at all, since one end (the alternative) is left "open" and the option "pro vita" always applies. The argument itself hasthe logical form of a *modus ponens *and it would be correct, by confirming the antecedent (premise), to conclude that life should be maintained, i.e., "there is doubt, so life should be maintained". On the contrary, although one could deny the antecedent, which would be to state that "there is no doubt", i.e. it is useless to try to maintain life, to imply that "life should **not **be maintained", would be a fallacy (which is the well known "fallacy of denying the antecedent"). It could be concluded that the logical structure of the argument itself does not offer much of a choice and the reasons for not maintaining the lives of the terminally ill patients should be looked for in the real world.

Therefore, we also wish to briefly consider the issue in the context of the real world, not in a simple logical framework. Indeed, it appears obvious that if one could only be sure as to when there **is **doubt and when **there is no **doubt, the above logical problems and probably all the problems of decision making would be solved. In the context of the real world, we suspect there is always some degree of doubt and one cannot create even a hypothetical situation where, when making a decision to terminate life, absolutely no doubt would exist. What is at issue here is, after all, not "doubt" itself, but the content of the doubt: whether the life of the terminally ill patient should be maintained or not. To decide this, one would have to reach a certain degree of certainty. While it appears to be of little importance to determine just how much one is certain that "doubt is present" (since in these circumstances, life maintenance measures would be undertaken anyway), it seems extremely important to determine exactly how certain it is that there is "no doubt" (since life cessation measures would be undertaken only if the certainty were to be absolute). Therefore let us examine the latter in some detail. However, we have to draw the reader's attention to the fact that events or entities which have a continuous nature if described as graded may carry a risk of arbitrary divisions that may be later overseen, and then a danger of a "slippery slope" [23] might appear as soon as some of the steps are challenged. The epistemic hierarchy offers an illustration of both kinds of difficulties.

If one considers the "Thirteen Steps" of the epistemic hierarchy (Appendix) [24] it is clear that one can, for example, place the proposition "there is no doubt" at position -4 (i.e. "it is **evidently false **that there is ***no ***doubt"; the emphasis is always ours) and still not be justified in discarding doubt completely. Some degree of doubt would persist even if one asserts, "it is **certainly false **that there is ***no ***doubt" (position -6). Here also we cannot say that doubt is excluded completely. Although one may make such an assertion, one can never be sure that it corresponds entirely to reality. The reason for this is that in real life we refer to the external world about which we can never have complete knowledge.

Indeed, the concept of certainty refers to our personal epistemological stance and not to an omniscient stance. Our personal conviction of how the world appears to be may not faithfully mirror how the world really is, and while one person may be certain about some matter, another may not be so certain. Regardless of how low the probability, even if it is infinitesimally small, its realization will always be theoretically possible – and doubt will forever lurk. If one were to act only then when in absolute certainty of what one is doing and when one is certain about the consequences of the acts, one would presumably never act. We act most of the time within large amounts of uncertainty and the results of our actions, sometimes, surprise us, or could even bring us, unexpectedly again, into opposition with our rigid legal system. The reality of the world and, as we have seen, logic, deceive us when we try to act according to our reason and in accord with nature. We will see that our humanly made systems of guidelines, more precisely, our legal system, can not be of much help either.

## Legal aspects

The rule "in dubio pro reo" – a merciful legal rule that favours the accused – does not suffer from the same shortcomings. In principle, tribunals apply law, not necessarily justice, while on the contrary, with the application of *in dubio pro vita*, justice is expected. Should *in dubio pro vita *become law, and if "in dubio" were to be interpreted as "reasonable doubt" (Appendix), this being a quite weak assertion, it would in all likelihood increase legal life cessation and make it more frequent, thereby increasing erroneous decisions. On the other hand, a more restricted interpretation of the rule would be "beyond any doubt" ("certain", Appendix), which has apparently occurred in Germany, where active life termination is practically forbidden.

However, the legal criterion "beyond reasonable doubt" suffers (in theory) from similar shortcomings of interpretation. Yet those who consider the rule "beyond reasonable doubt" are typically a collective (judges, jury) and not a single person (as is the case for medical personnel). Judges are not liable if wrong, although their decisions may be challenged some later instance. If a judge's decision is challenged and overruled, she/he suffers no legal consequence. On the contrary, a physician's decision to terminate life may be legally challenged and the physician may be held responsible. Death penalty decisions are perhaps the most similar to a physician's decision to terminate life, but these decisions have one major difference. Apart from what has already been mentioned about judicial decisions, death penalty decisions can (almost) always be reconsidered with an appeal, not only in theory but also in practice. Whereas an initial decision by a physician to terminate human life is without appeal and is usually acted upon immediately.

## Natural and positive justice

Indeed, it may appear to a reader that we maintain that giving the force of law to such decisions may automatically introduce injustice. People are, nevertheless, often inclined to identify law with justice. What is often observed is not that "justice has been done" but that "law has been applied" and consequently then it is seen (!) as "justice is being done".

However, if examined with more scrutiny, it is not difficult to realize that law is an expression of the society and of the political will and not only of how justice is appreciated at a given point of time in history. That laws could be unjust has been common experience in the past and is frequent experience even today. For example, the opponents of capital punishment maintain that the death penalty is in fact unjust in principle. However, we do not always agree with the cruel punishment of criminals that is a normal, lawful procedure in some countries. Indeed, the distinction between law and justice was recognized very early [25,26]. Related to the context here, it is maintained that if theoretical ambiguities are present, as they are with the rule *in dubio pro vita*, then the possibilities of applying the law without promoting our intuitive idea of justice, may be increased.

On the one hand the physician understands that she/he has to follow morally acceptable reasons. On the other hand, in theory, she/he understands that to support life is always a valid and defendable choice. The attending physician is not in a position to objectively determine when there is "no doubt" and has only one valid and secure option, to "go for life". Consequently, as a legal principle, *in dubio, pro vita *would be empty – i.e. it offers little other than to always support life. The proper interpretation of that rule is that doubt may always obtain so the physician does not have a choice. Some unfortunate physician who might decide on moral and medical grounds that there is "no doubt" that someone's life should be terminated in a passive or an active way (it being given that legislation would permit it), may find himself in a perilous situation if just one sceptic were to challenge that decision – and that sceptic happened to be her/his judge. If the rule is ambiguous, giving it the force of law and then punishing someone for not observing that law would not be just. To promulgate a law that permits the false belief that there is a choice – is unjust.

## Summary

It has been argued in the present article that the guidelines for life termination decisions should be carefully reconsidered. The moral rules should probably be given a slight indeterministic turn in the sense of giving the advantages to the judgments made according to the actual circumstances, over and above some a priori established strict law-like set of regulations.

We contend that the rule, *in dubio pro vita *which has gained popularity, at least in Europe, would be of no value as law since it would be open-ended and always allow for doubt. According to this "law" – if applied prudently – the decision to take measures that maintain human life would always be the single valid choice. Preservation of this rule may be encouraged, but giving it the force of law might represent a risk for medical personnel who choose not to support human life in some otherwise morally, medically and existentially uncontroversial circumstance. In other words, such a decision may, at least in theory, be challenged even if the termination of life support is fully morally justified.

## Competing interests

The authors declare that they have no competing interests.

## Authors' contributions

DP initiated the project and wrote the manuscript. ChL and MW discussed initially the project, read and corrected various versions of the manuscript and helped develop arguments.

## Appendix

The 13 Steps of the epistemic justification hierarchy, according to Chisholm R. [24]. This is a spectrum that describes the degree of evidence supporting some proposition, from 'certainly true' to 'certainly false"'.

6. Certain

5. Obvious

4. Evident

3. Beyond Reasonable Doubt

2. Epistemically in the Clear

1. Probable

0. Counterbalanced

-1. Probably False

-2. In the Clear to Disbelieve

-3. Reasonable to Disbelieve

-4. Evidently False

-5. Obviously False

-6. Certainly False

## About the authors

DP (MD) is Research Director at the Department of Anesthesiology and Intensive Care Medicine at the University of Greifswald, Germany. His interest is focused on fundamental muscle research related to circulation in experimental sepsis, and on bronchial hyperreactivity. Additionally his research involves medical ethics, philosophy of science and scientific method, as well as history of medicine.

ChL (MD) is Professor of Anesthesiology at the Department of Anesthesiology and Intensive Care Medicine at the University of Greifswald, Germany. His main interest is clinical and basic sepsis research, mainly centered on microcirculation. He is also interested in ethical issues related to the end-of-life care.

MW (MD), Professor of Anesthesiology and intensive Care Medicine and chief of the Department of Anesthesiology and Intensive Care Medicine at the University of Greifswald, Germany. His research interests focus on Intensive Care Unit management, hospital management, but also include cultural and ethical aspects of biomedical science and of the end-of-life care.
